# Common and Unique Genetic Background between Attention-Deficit/Hyperactivity Disorder and Excessive Body Weight

**DOI:** 10.3390/genes12091407

**Published:** 2021-09-13

**Authors:** Monika Dmitrzak-Weglarz, Elzbieta Paszynska, Karolina Bilska, Paula Szczesniewska, Ewa Bryl, Joanna Duda, Agata Dutkiewicz, Marta Tyszkiewicz-Nwafor, Piotr Czerski, Tomasz Hanc, Agnieszka Slopien

**Affiliations:** 1 Department of Psychiatric Genetics, Poznan University of Medical Sciences, 60-806 Poznan, Poland; kbilska@ump.edu.pl (K.B.); joanna.duda@ump.edu.pl (J.D.); pczerski@ump.edu.pl (P.C.); 2 Department of Integrated Dentistry, Poznan University of Medical Sciences, 60-812 Poznan, Poland; paszynska@ump.edu.pl; 3 Institute of Human Biology and Evolution, Faculty of Biology, Adam Mickiewicz University, 61-614 Poznan, Poland; paula.mamrot@amu.edu.pl (P.S.); ewa.bryl@amu.edu.pl (E.B.); tomekh@amu.edu.pl (T.H.); 4 Department of Child and Adolescent Psychiatry, Poznan University of Medical Sciences, 60-572 Poznan, Poland; adutkiewicz@ump.edu.pl (A.D.); mtyszkiewicz@ump.edu.pl (M.T.-N.); agaslopien@ump.edu.pl (A.S.)

**Keywords:** case-control association study, whole exome sequencing, attention-deficit/hyperactivity disorder, excessive body weight

## Abstract

Comorbidity studies show that children with ADHD have a higher risk of being overweight and obese than healthy children. This study aimed to assess the genetic alternations that differ between and are shared by ADHD and excessive body weight (EBW). The sample consisted of 743 Polish children aged between 6 and 17 years. We analyzed a unique set of genes and polymorphisms selected for ADHD and/or obesity based on gene prioritization tools. Polymorphisms in the *KCNIP1, SLC1A3, MTHFR, ADRA2A*, and *SLC6A2* genes proved to be associated with the risk of ADHD in the studied population. The *COMT* gene polymorphism was one that specifically increased the risk of EBW in the ADHD group. Using the whole-exome sequencing technique, we have shown that the ADHD group contains rare and protein-truncating variants in the *FBXL17, DBH, MTHFR, PCDH7, RSPH3, SPTBN1*, and *TNRC6C* genes. In turn, variants in the *ADRA2A, DYNC1H1, MAP1A, SEMA6D*, and *ZNF536* genes were specific for ADHD with EBW. In this way, we confirmed, at the molecular level, the existence of genes specifically predisposing to EBW in ADHD patients, which are associated with the biological pathways involved in the regulation of the reward system, intestinal microbiome, and muscle metabolism.

## 1. Introduction

Increasing attention has been devoted to the comorbidity between attention-deficit/hyperactivity disorder (ADHD) and overweight/obesity in the last few years. Researchers have observed higher body weight or body mass index (BMI) among children and adolescents with ADHD than growth charts or control groups of individuals without ADHD suggest. Additionally, a higher percentage of overweight and/or obesity was noted in children and adolescents with ADHD. The association between ADHD and overweight/obesity among children or adolescents has been demonstrated in clinical and epidemiological studies (see reviews: [1,2,3,4]). A similar relationship between ADHD and obesity was found in epidemiological studies in adults [5,6]. ADHD is also diagnosed more often in clinically obese children [7], adolescents [8], and adults [9,10]. Several mechanisms have been proposed to explain the possible link between ADHD and obesity. From a theoretical point of view, it is possible that:

(1) Obesity and/or obesity-related factors (e.g., impaired sleep breathing) lead to symptoms of ADHD;
(2) ADHD increases the risk of obesity (e.g., impulsivity and deficits in executive functions favor inappropriate eating behavior);
(3) ADHD and obesity share common biological dysfunctions (e.g., disorders of the dopaminergic system and reward deficiency syndrome) [11,12,13,14].

The basis of biological dysfunction comprises the genetic risk factors, which may be partially shared for ADHD and obesity. Data from different types of research (clinical, case studies, animal models studies) suggest that shared genes for both diseases are: dopamine D2 and D4 receptor genes (DRD2, DRD4), brain-derived neurotrophic factor gene (BDNF), and melanocortin-4-receptor gene (MC4R) [12]. The genome-wide association study (GWAS) of a German population identified a directionally consistent association of two obesity risk alleles with ADHD: SNP rs206936 (located in intron 1 of NUDT3—a member of the Nudix protein family) and SNP rs6497416 (located in an intronic region of GPRC5B—a member of the superfamily of G protein-coupled receptors GPCR) [15]. It is essential to use the endophenotype/subphenotype strategy for much greater power in identifying the risk genes. The specific subphenotype is closer to the actions of the genes related to the selected traits than disease status alone. The individual differences in presenting obesity in response to ADHD could be genetic, so the more we know about the genes involved, the more individualized the treatment can be. This knowledge could improve not only patient outcomes but also increase long-term compliance to treatment and diet. Additionally, coexisting obesity can be used as “traits” to select a specific subgroup of patients to perform association analysis. According to this knowledge, the fat and obesity-associated transcript 3 gene (FTO α-ketoglutarate dependent dioxygenase, *FTO*) seems to be a good candidate gene spanning the biological mechanism responsible for increased food intake in ADHD patients [16,17]. Even if the ADHD risk genes have small effect sizes in the population, their identification may still be highly relevant clinically because gene variants may explain most of the heritability in individual patients. Moreover, understanding their functions and the pathways between each gene and behavior may translate into improved diagnosis and treatment strategies. Thus, we propose comprehensive genetic analysis of Polish ADHD and non-ADHD (healthy control) subjects.

Given the above, the purpose of this study is to investigate whether:

- Polymorphisms of a unique set of genes selected based on gene prioritization tools are associated with the risk of ADHD;
- There are relationships between the studied polymorphisms and overweight and obesity named excessive body weight (EBW);
- The use of whole-exome sequencing allows for the determination of common and unique as well as rare and truncating protein variants between ADHD and EBW.

We will also try to discuss potential mechanisms linking the identified genetic variants with the biological pathways observed in the ADHD group with EBW.

## 2. Materials and Methods

### 2.1. Ethical Statement

The study used data collected during a project carried out in the years from 2017 to 2020. The study was approved by the Ethics Committee, University of Medical Sciences in Poznan (resolution no. 542/14 from 6 December 2014). All study participants were Caucasians of Polish origin. Parents or legal guardians gave written informed consent for the participation of their children in the study. Before the examination, we explained the purpose and principles of the study to all of the children and asked them to express their opinions.

### 2.2. Participants

ADHD group: Patients diagnosed or preliminarily diagnosed with ADHD from the western part of Poland were invited to participate in the project through advertisements/information published, i.e., in psychological and pedagogical counseling centers, mental health outpatient clinics for children and youth and the university website. Willing parents and their children were invited to the Children and Adolescents Psychiatry Department, Poznan University of Medical Science (PUMS), where the recruitment took place. A trained group of child psychiatrists and psychologists verified the diagnosis based on the interview, observation and provided medical documentation. The main inclusion criterion was the ADHD diagnosis confirmed by specialist consultation according to ICD-10 and DSM-5 criteria with using polish version of the diagnostic structured interview for ADHD and hyperkinetic disorder [18], K-SADS-PL [19] and Conners’ Rating Scales (teacher and parent version) [20]. Out of 261 reports, 17 did not come to the appointment, 2 families withdrew their consent to participate in the study, and in 16 children the diagnosis was not confirmed or other disorders were found (i.e., obsessive-compulsive disorder (OCD), autism spectrum disorder (ASD), emotional dysregulation (ED) or neurological disorders). Finally, the study group consisted of 226 children with confirmed ADHD diagnosis, with ages ranging from 6 to 17 years (10.4 ± 2.6; 204 (90.2%) boys and 22 (9.8%) girls)).

Non-ADHD group: The voluntary non-ADHD control group was recruited in primary schools in Poznan, Poland. The inclusion criterion was a lack of distinct symptoms of ADHD (MINI-Kid [21], Conners’ Rating Scales-Revised [22] or diagnosis of ADHD among firs- degree relatives). Initially, the parents of 519 children agreed to participate in the project. Despite an initial declaration, fifteen families did not participate in the project for unknown reasons; three withdrew their consent; seven changed schools during the year; twelve were absent during the study. Finally, 482 children were included into the control group, with ages ranging 6–12 years (9.0 ± 1.3; 250 boys (51.8%) and 232 (48.2%) girls).

Exclusion criteria for both groups were as follows: schizophrenia, bipolar disorder, organic brain dysfunction (e.g., epilepsy, serious trauma and infections of central nervous system), any somatic disorders and treatment which might affect behavior, cognitive functions or growth. Inclusion and exclusion criteria for both groups are shown in Table 1.

### 2.3. Anthropometric Data

Trained research staff collected the weight and height of participants using standard anthropometric procedures. Obtained data were used to calculate BMI (kg/m^2^). Underweight, healthy weight, overweight, and obesity were diagnosed with the International Obesity Task-Force criteria (IOTF) based on children’s BMI. The reference values of the IOTF were used to take into account the dynamics of children’s development, age, and gender. Criteria were normalized for children aged 2–18 years with an accuracy level of up to half a year [23,24].

### 2.4. Biological Material Collection and DNA Extraction

Saliva samples were collected using the Oragene DNA^®^ (OG-500) self-collection kit (DNA genotekTM, Ottawa, Canada), following the manufacturer’s instructions. Extraction of DNA from saliva samples was performed using the prepIT^®^ L2P manual protocol to purify DNA from a 4 mL sample [25]. In patients who failed to collect their saliva, the blood samples were collected by a trained nurse using BD Vacutainer^®^ K3 EDTA collection tubes. The DNA was extracted from 5 mL of EDTA anticoagulated whole blood using the salting-out method [26].

In both methods, the DNA comes from leukocytes. The methods used are equivalent and do not interfere with single nucleotide polymorphism (SNP) and high-throughput analyses [27].

The Nanodrop 2000c Spectrophotometer (Thermo Fisher ScientificTM, Wilmington, NC, USA) was used to quantify and assess the quality of extracted DNA. Purified DNA were stored at −80 °C until further analysis.

### 2.5. Gene and SNP Selection

To select genes predisposing to ADHD and obesity, we used bioinformatics methods of prioritizing genes with the highest probability of their involvement in the development of the studied phenotypes [28]. This strategy is based on creating a ranking of the genes identified in silico by classifying them according to their potential importance in developing the analyzed trait [29]. According to decision tree grouping tools that best suit selected candidate polymorphism based on genome data, we select two tools also because of their intuitive use and open access [30]. GeneProspector searches for evidence about human genes in relation to diseases, other phenotypes, and risk factors and selects and prioritizes candidate genes by using a literature database of genetic association studies (not supported) [31] and PolySearch extracts and analyzes relationships between diseases, genes, mutations, drugs, pathways, tissues, organs, and metabolites in humans by using multiple biomedical text databases [32]. In addition, we supplemented the list of studied genes and polymorphisms with significant results of GWAS analyses. The subset of SNPs of interest covering the desired candidate genes are presented in Table S1.

### 2.6. Genotyping

The polymorphisms have been identified with use of KASP™ genotyping assays provided by the LGC genomic laboratory (LGC, Hoddesdon, UK). In summary, KASP genotyping assays are based on competitive allele-specific multiplex PCR. Allelic discrimination is based on the competitive binding of two allele-specific primers labelled with FAM™ or HEX™ dye. The detailed described protocols can be found in the Biosearchtech producers user guide [33,34,35].

The SNPs that failed in the KASP multiplex assay were genotyped using TaqMan SNP Genotyping assays (Applied Biosystems, Foster City, CA, USA) and HOT FIREPol Probe qPCR Mix Plus (ROX) (Solis BioDyne, Tartu, Estonia) according to the manufacturer’s instructions. The amplification was done in AbiPrism 7900HT Real-Time PCR System. Data acquisition and analysis were performed using the allelic discrimination analysis module in SDS v2.4 software (Applied Biosystems, Singapore, Singapore). As a quality control measure, negative controls and ~5% of samples were genotyped in duplicate to check genotyping accuracy. Information about the analyzed SNPs, primers and assays is shown in Table S1.

### 2.7. Statistical Analysis

The results were expressed as numbers and percentages, mean ± standard deviation and median, as appropriate. Statistical analyses were conducted in Statistica v13.3 (StatSoft, Cracow, Poland), VassarStats (http://vassarstats.net/ (accessed on 3th August) and G*Power 3.1 (University Dusseldorf, Dusseldorf, Germany) software. The differences in the clinical and sociodemographic features between ADHD and non-ADHD were compared using the non-parametric chi-square test. A multiple linear regression analysis was used to assess the influence of age and sex on other analyzed clinical and molecular variables. Spearman’s rank correlation was used to detect the relationship between variables. Genotype and allele frequencies were compared using the chi-square test and Fisher exact test, respectively. All analyzed polymorphisms were at Hardy–Weinberg equilibrium. All tests were two-tailed, and α was set at 0.05 (as we compared two groups at the same time only).

To determine a priori minimal sample size, we used G*Power 3 Software [36,37]. Given α = 0.05; power (1–β) = 0.8; a medium effect size, total desired sample size was as follows: n = 108 for genetic analyses and chi-square test (w = 0.3); n = 68 for multiple regression analysis (f2 = 0.15); and n = 400 (allocation ratio N1/N2 = 0.5) for correlation (n = 133 for ADHD and n = 267 for non-ADHD).

Therefore, the study included an adequate sample size, and the statistical power was appropriate to detect significant differences in the studied groups.

### 2.8. WES Sequencing

**Figure 1:**
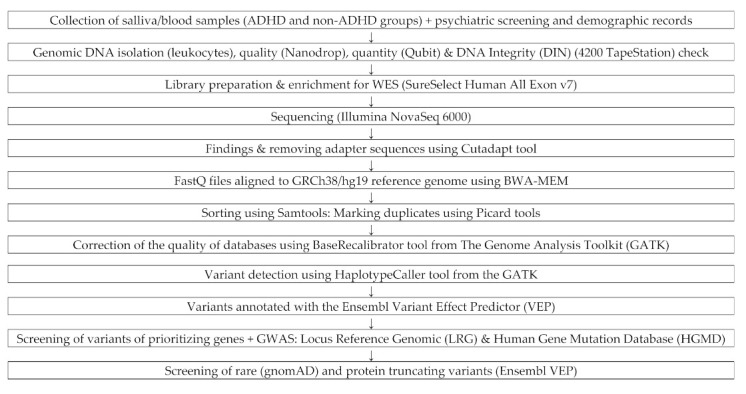
Diagram of WES Sequencing: laboratory experiment and bioinformatics pipeline.

Whole-exome sequencing was carried out as previously described [38]. Paired-end, indexed libraries for Illumina sequencing were prepared from the DNAs of the 50 ADHD and age- and gender-matched non-ADHD children. No other selection criteria were used. Library enrichment for WES was conducted using SureSelect Human All Exon v7 from Agilent Technologies (Santa Clara, CA, USA) [39]. Enriched samples were sequenced using an Illumina NovaSeq 6000 platform (San Diego, CA, USA) [40]. Mean coverage of the sequences was 50× on target. The study design, laboratory experiment and bioinformatics pipeline based on the modified protocol of Tombacz et al. is presented in Figure 1 [41].

### 2.9. Bioinformatic Analysis

In the first stage of the analysis of the results, the adapter sequences specific for the type of library being prepared were removed from the raw sequences using the Cutadapt software (version 1.14) [42]. The analysis of the quality of the readings was performed with the FastQC software (version 0.11.8) [43]. The mapping to the human genome reference sequence in the GRCh38/hg19 version was performed using the bwa-mem algorithm from the BurrowsWheeler Aligner package (BWA, version 0.7.15-r1140) [44]. Sorting and other auxiliary operations on BAM files were performed with Samtools (version 1.3.1) [45], and marking duplicates with Mark Duplicates from Picard (version 2.20.4) [46]. Correction of the quality of databases was performed with the use of the BaseRecalibrator tool from the Genome Analysis Toolkit (GATK, version 4.0.10) [47]. Variant detection was performed with the HaplotypeCaller tool from the GATK package (version 4.0.10) [47], and variants were annotated with the Ensembl Variant Effect Predictor (VEP, version 99) [48].

The main objective was to analyze the variants in the genes associated with ADHD and obesity described in the Gene and SNP selection section (Table S1) and supplemented with the genes selected additionally based on updated literature reports covering rare and protein shortening variants (STOP codon, frameshift) (Table S2) [49,50].

For the list of genes, transcripts were selected for further analysis based on the following databases: Locus Reference Genomic (LRG) [51], Human Gene Mutation Database Professional 2020.2 [52], and for single genes not present in the above databases, the NCBI transcript according to the HGNC database [53]. As a result, a set of 672 unique variants was obtained (Table S3). The next step was to select variants with the IMPACT value (derived from Ensembl VEP annotation [48]) equal to HIGH or MODERATE, which corresponds to the consequences such as stop_gain, frameshift, stop_lost, inframe_insertion, inframe_deletion, missense_variant [54]. The next step was to narrow down the variants to rare (rate threshold 0.009%) and very rare (0.0009%) using the gnomAD v2.1 NFE (Exomes) previously described [55,56]. After such a narrowing down, a list was obtained, of 48 variants in 30 genes for rare variants (Table S4) and 25 variants in 19 genes for very rare variants (Table S5), respectively.

## 3. Results

### 3.1. Demographic Characteristic

The clinical characteristics of ADHD and non-ADHD children are presented in Table 2.

The study groups differed in mean age and gender distribution. Patients with ADHD were older than non-ADHD, and boys predominated in this group.

Children with ADHD were also characterized by: more frequent living in rural areas, younger age of parents at the time of childbirth, lower level of parents’ education, and lower socioeconomic status. Moreover, in this group, the occurrence of comorbid disorders, preterm labor, assisted delivery, lower birth weight, and Apgar score compared to the group of non-ADHD children were more frequent. The reported features are consistent with previous observations [4,57]. We did not observe any significant differences between the ADHD and the non-ADHD group in the case of weight status.

To control for sex and age as confounding factors for genotype frequencies, we performed the multiple regression analysis. This analysis showed no significant effects of age (*p* = 0.550) or sex (*p* = 0.294) on molecular variables (F(2, 571) = 0.630; *p* = 0.532; R^2 = 0.002). Thus, in further analyses, groups were not subdivided according to age and sex of participants.

### 3.2. Genotyping

From the list of 35 selected polymorphisms, we were able to determine genotypes in 26 cases. Genotype and allele frequencies in ADHD and non-ADHD groups are presented in Table 3.

Two SNPs (rs3772475/*FHIT* gene and rs6283/*DRD5* gene) were non-polymorphic in studied populations. Two others (rs518147 and rs3813929 in the *HTR2C* gene) presented deviation from the Hardy–Weinberg equilibrium law. Case-control comparisons between ADHD and non-ADHD groups showed an association as follows:

- ADRA2A—adrenoceptor α 2A gene (rs1800544 *p* = 0.018 for alleles),
- KCNIP1—potassium voltage-gated channel interacting protein 1 gene (rs1541665 *p* = 0.044 for genotypes; *p* = 0.015 for alleles),
- MTHFR—methylenetetrahydrofolate reductase gene (rs1801131 *p* = 0.013 for genotypes),
- SLC1A3—solute carrier family 1 member 3 gene (rs1049522 *p* = 0.028 for genotypes; *p* = 0.022 for alleles),
- SLC6A2—solute carrier family 6 member 2 gene (rs5569 *p* = 0.029 for alleles).

The shown nominal associations did not survive the Bonferroni correction taking into account the analyzed number of genes (threshold *p*-value= 0.002).

We did not find any significant differences in genotype and allele frequencies between the compared group of patients and controls for other analyzed polymorphisms.

In the second stage of the analysis, we attempted to check whether there is an association of the studied polymorphisms with excess body weight in the examined ADHD and non-ADHD subgroups. Overweight and obese children were separated into a subgroup defined as excessive body weight (EBW) compared to the group of children with normal body weight (NBW). The results of Spearman’s rank correlation analysis are presented in Table S6 in the supplementary materials. The relationship between EBW polymorphism and *PIK3CG* (phosphatidylinositol-4,5-bisphosphate 3-kinase catalytic subunit γ) was indicated: rs12667819 (*p* = 0.014) in the non-ADHD group; *COMT* (catechol-O-methyltransferase): rs4680 (*p* = 0.048) in the ADHD group. For the polymorphisms selected in this way, the frequencies of genotypes and alleles were compared using the ch2 and Fisher test, respectively.

More frequent occurrence of the GG genotype (*p* = 0.019) and the G allele (*p* = 0.005) of the *PIK3CG* polymorphism: rs12667819 was confirmed in children with EWB in the non-ADHD group. On the other hand, in the ADHD group, the A allele of the *COMT* polymorphism: rs4680 was more common.

### 3.3. Rare and Protein-Truncating Variants

After conducting Whole Exome Sequencing (WES), the search was narrowed down to rare variants, focusing on variants damaging proteins in a unique selected group of genes. The last step was to use Fisher’s exact test to answer the question about the relationship between the occurrence of variants in the selected genes and the phenotype under study, i.e., the diagnosis of ADHD and ADHD and EBW, respectively. Statistical analysis was performed in the R environment using Fishers exact test.

We looked for variants present in the ADHD group but not present in the non-ADHD group (Table 4A). We identified 13 genes with identified variants (*ADRA2A, DBH, DYNC1H1, FBXL17, MAP1A, MTHFR, PCDH7, RSPH3, SCN2A, SEMA6D, SPTBN1, TNRC6C, ZNF536*). Despite the relatively small study group and the analysis of rare variants in the *FBXL17* gene (F-box and leucine rich repeat protein 17), it was possible to confirm a statistically significant difference in the frequency of variants in this gene between ADHD patients and healthy controls (*p* = 0.033).

Similarly, we searched for variants in the ADHD with EBW vs. not detected in the non-ADHD with NBW group (Table 4B). We identified six genes with variants (*ADRA2A, DYNC1H1, FTO, MAP1A, SEMA6D, ZNF536*) present in the ADHD with EBW group, although without a significant difference compared to the control group.

**Figure 2:**
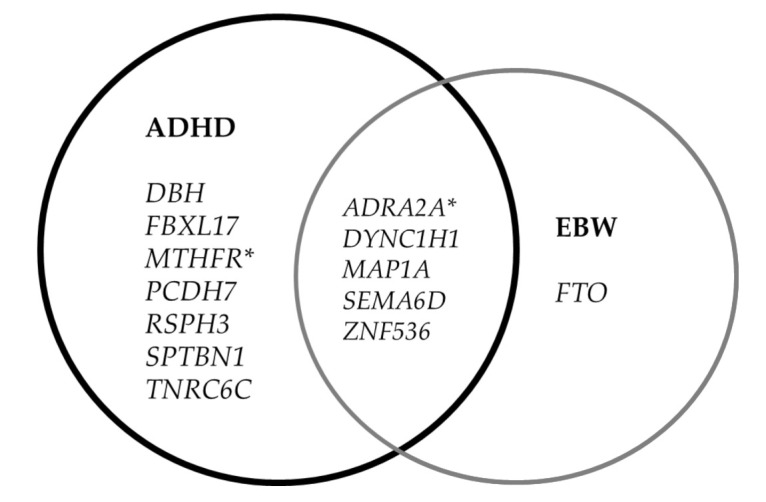
Venn diagram representing common and shared genes between ADHD and EBW. * genes associated with ADHD by SNP polymorphisms.

In order to identify common and distinct genes for ADHD and EBW, we used the Venn diagram (Figure 2).

## 4. Discussion

We planned initially to include children aged 6–12 years only. In those children with ADHD, due to the small number willing to participate in the study, we extended the age range to 17 years. We took advantage of the fact that age did not affect the gene polymorphisms that remain unchanged throughout life. Although it is now assumed that the prevalence of ADHD in both sexes is similar, due to the type and severity of symptoms, boys dominate in clinical trials [58], including in our study [59]. Regression analysis confirmed no significant influence of age and sex on genetic variables. As BMI is dependent on gender and age, in assessing overweight and obesity we used a diagnostic method adjusted for age and gender. The most prevalent presentation/subtypes of ADHD were the combined type (75.7%), followed by the ADHD inattentive (17.3%) and ADHD hyperactive-impulsive types (7.0%), which is consistent with earlier observations [60,61]. Similarly, in other recorded characteristics such as comorbidity [57] or the socioeconomic status of the family [62], our patients did not differ from the previously observed clinical groups [63].

### 4.1. Genes and Polymorphisms

Classic association studies included a unique set of genes and polymorphisms selected on the basis of the results of gene prioritization studies for ADHD and/or obesity supplemented with GWAS results. Polymorphisms in the *KCNIP1* (rs1541665) [64], *SLC1A3* (rs1049522) [65], *MTHFR* (rs1801131) [66], *ADRA2A* (rs18005) [67] and *SLC6A2* (rs5569) [68,69] genes were associated with ADHD risk, which is in line with previous reports.

The polymorphism of the COMT gene (rs4680) was explicitly associated with EBW in the ADHD group. The study results indicated that, in the ADHD group, there were at least partially separate pathways that increased the risk of excess body weight in the group of ADHD patients. Our results support the hypothesis of the participation of the dopaminergic system in ADHD and reward sensitivity [70]. It was found that participants with high levels of ADHD symptoms and genetic profiles associated with greater dopaminergic activation in crucial brain reward areas were more likely to engage in hedonic eating and, as a result, have a higher BMI [2].

In our search for genes that specifically increase the risk of obesity in the course of ADHD, we found that the polymorphism of the PIK3CG gene (rs12667819) was associated with the risk of excess body weight in the control group. The knowledge available to us shows that this is the first molecular evidence supporting the observations that PI3Kγ activity in leukocytes promotes adipose tissue inflammation and early-onset insulin resistance during obesity. The detailed biochemical mechanism was presented by Breasson et al. [71].

### 4.2. Rare and Protein-Truncating Variant (PTV)

While searching for rare and PTV variants related to ADHD risk in the studied population, we were able to select a group of 13 genes. The *MTHFR* and *ADRA2A* genes were also crucial in our classic SNP association analysis. For the *MAP1A, ZNF536, SPTBN1, TNRC6C* and *SCN2A* genes, we replicated the results obtained by Satterstrom et al. [50]. In turn, in the case of the *SEMA6D* and *FBXL17* genes, we replicated the results of Rovira et al. [49]. The results confirm the necessity to continue searching for robust biological markers among rare features previously overlooked before the era of high-throughput research.

In turn, in looking for variants related to EBW, we identified six relevant genes (*ADRA2A, DYNC1H1, FTO, MAP1A, SEMA6D, ZNF536*). Using a Venn chart, we selected genes common and different for ADHD and EBW. This included seven genes specific for ADHD. Especially intriguing was the *FBXL17* gene (F-box and leucine rich repeat protein 17) coding member of the F-box protein family, which acts as protein-ubiquitin ligases [72]. For the first time, the *FBXL17* gene was identified in the analysis of interaction networks enriched among genes with SNP coding that correlate with bacterial taxa abundance in humans as presented by Blekhman et al. [73]. In turn, Martinis-Silva et al. demonstrated that, specifically, the *FBXL17* gene as a host gene is associated with modulating the composition of the gut microbiome and may influence ADHD susceptibility [74]. Furthermore, several studies have shown that the gut microbiome may play a role in ADHD [75,76]. Host genetic variants may prevent the attendance of specific bacterial species, considerably altering the diversity of microorganisms. However, it is still unclear how the *FBXL17* gene would influence the composition of the gut microflora and contribute to the development of ADHD risk [77].

Although an association between obesity-related *FTO* gene and ADHD was previously mentioned [16], the *FTO* gene showed only an EBW relationship in our analysis. This observation was in line with previous reports that the *FTO* region harbors the strongest genetic association with obesity, yet the mechanistic basis of this association remains elusive [78,79]. It is indicated that in the case of ADHD, variants of this gene may play a protective role [80].

The group of genes common for ADHD and EBW (*ADRA2A, DYNC1H1, MAP1A, SEMA6D, ZNF536*) indicates that, in ADHD, we may have to deal with biological pathways leading to excess body weight not found in the classic and direct causal relationship. An example of this is the research results presented by Amare et al. [81], which revealed 24 potential pleiotropic genes that are likely common to cardiovascular, metabolic, and mood disorders. Among them are also genes identified in our study as common to ADHD and EBW, including ADRA2A, a cell-surface G-protein-coupled receptor (GPCR) for catecholamines that controls various physiological functions, such as the modulation of neuron transmission, smooth muscle contraction, energy homeostasis thermoregulation, and the metabolism of glucose and lipids [82]. This may also affect the dimensions of temperament and mood, which translates into how food is consumed [83], thus linking the predisposition to EBW in children with ADHD.

Our current and previous discoveries emphasize the critical role of the muscular system and its metabolism, often overlooked in molecular studies even though hyperactivity is a crucial symptom of ADHD. The *DYNC1H1* gene encodes dynein, which belongs to a group of microtubule-activated ATPases that function as molecular motors [84]. Mutations in this gene are mainly associated with neuromuscular diseases [85]. However, there is also a case presenting mutation in this gene in ADHD [86]. This result partially supports the earlier discovery of Filer et al., who performed a GWAS to identify genes contributing to motor coordination problems, and among the highest-ranked genes were *MAP2K5*, involved in restless legs syndrome, and *CHD6*, causing motor coordination problems in mice [87]. Hyperactivity is associated with an excessive increase in the intensity of muscle work and an increase in energy expenditure on various activities (e.g., drilling, aimlessly walking, gestures) [88]. In addition, most children who are diagnosed with ADHD also have motor problems, especially motor inhibition problems and high muscle tone [89]. Incorrect and uneven muscle work may contribute to metabolic disorders and the accumulation of energy reserves (especially at rest, during sleep), leading to obesity.

The other three genes identified as common to ADHD and EBW are *MAP1A, SEMA6D*, and *ZNF536*, for which the common denominator is the involvement in neurogenesis.

The *SEMA6D* gene encodes semaphoring, which is also involved in the orientation of axonal growth [90]. In the ADHD animal model (Lister hooded rats), the decreased expression of Sema6d gene mRNA was found [91]. In the body mass gain animal model (female piglets), differential expression of the SEMA6D gene was observed as a consequence of early exposure to antibiotics linked with microbiota changing and increasing the risk of overweight or obesity later in childhood [92].

The *MAP1A* gene encodes a protein that belongs to the microtubule-associated protein family [93]. An analysis of the rat brain proteome revealed significant changes that indicate that a combination of the Western diet and stress exposure may lead to neuronal function and signaling impairments. Researchers working on the animal model proved the down-regulation of the proteins involved in neurotransmitter secretion and learning and memory mechanisms, indicating the role of the Map1a gene in both processes [94].

The *ZNF536* gene encodes Zinc Finger Protein 536, probably presenting transcription repressor activity [95]. Little is known about the functional significance of this gene, and it is difficult to associate its action with both ADHD and/or obesity. So far, only one study has found altered variants of this gene in patients presenting both ADHD and ASD symptoms [50].

Observations conducted over the past four decades confirm the systematic increase in the prevalence of overweight and obesity among children and adolescents in many countries in Europe and around the world [96]. The same trend also applies to children in Poland [97]. The percentage equalization of children with excess body weight in the control group and ADHD may result from several environmental factors that escalate from generation to generation (e.g., stress, the pace of life, easy access to relatively cheap and highly processed food). These, in turn, change eating habits and consequently lead to EBW in children not burdened with other developmental disorders [98]. The growing problem of overweight and obesity among children shows how important it is to discover genes that, on the one hand, create a predisposition to obesity and, on the other hand, are altered in expression under the influence of unfavorable environmental factors. In this regard, a stable group of patients with ADHD and EBW seems to be the excellent reference point for extrapolating to the entire population in the future.

### 4.3. Limitations

The total sample size was small, though comparable to other available studies, and achieved a strength of 80% for SNP association studies. However, to analyze the rare variants identified in the WES technique, the study group should be gradually expanded. We performed the analysis on a carefully selected unique group of genes that does not exclude the participation of others that could have some involvement. In further studies, it is worth counteracting gender imbalance and not omitting girls less frequently participating in clinical trials due to the milder phenotype of the disease. Considering the relatively low number of overweight children with ADHD, the relevance of the genetic variants described to increase the risk of EBW in children with ADHD needs to be confirmed in a larger cohort, including more overweight ADHD patients in future studies.

## 5. Conclusions

Our review provides insight into the common and different biological mechanisms of ADHD and excess body weight. Classic association studies included a unique set of genes and polymorphisms selected based on gene prioritization analysis for ADHD and/or obesity supplemented by GWAS results. Polymorphisms in the *KCNIP1, SLC1A3, MTHFR, ADRA2A*, and *SLC6A2* genes proved to be associated with the risk of ADHD in the studied population. The *COMT* gene polymorphism was a gene that specifically increased the risk of EBW in the ADHD group. In the analysis of the WES research, we focused on assessing the occurrence of rare and protein-truncating variants in the ADHD group. We have shown that the ADHD group includes rare and PTV variants in the *FBXL17, DBH, MTHFR, PCDH7, RSPH3, SPTBN1*, and *TNRC6C* genes.

On the other hand, variants in the *MAP1A, SEMA6D, ZNF536, ADRA2A* and *DYNC1H1* genes were specific for ADHD with EBW. In this way, we confirmed the existence of genes specifically predisposing to EBW in ADHD patients, which are associated with different biological pathways leading to excess body weight as an alternative to the classic and direct cause-and-effect type of relationship (i.e., the reward system, intestinal microbiome, and muscle metabolism). The presented results confirm the need to continue searching for robust biological markers, in both common and rare variants.

## Figures and Tables

**Table 1:** Inclusion and exclusion criteria for ADHD and non-ADHD groups.

Criteria for Inclusion into the ADHD Group	Criteria for Inclusion into the Non-ADHD Group	Criteria for Exclusion from ADHD and Non-ADHD Groups
Children of both sexes aged 6–17	Children of both sexes aged 6–12	Children with disorders of central nervous system (e.g., epilepsy, serious injuries, and CNS infections)
Children with diagnosed ADHD in accordance with ICD-10 and DSM-V diagnostic criteria (diagnosis confirmed by two independent psychiatrists based on a standardized and structured interview)	Lack of mental disorders—assessment with the use of MINI-Kid questionnaire	Co-existing: schizophrenia, bipolar affective disorder, any serious somatic disorders
Clinically significant ADHD symptoms lasting over 6 months	Parent or legal guardian approval	Chronic somatic diseases
Children without hereditary mental disorders (first-degree relatives)		Persistent pharmacotherapy, hormonotherapy
Parent or legal guardian approval		Lack of acceptance from parents or legal guardians

ADHD—Attention-Deficit/Hyperactivity Disorder; ICD-10—International Statistical Classification of Diseases and Related Health Problems (10th edition); DSM-V—Diagnostic and statistical manual of mental disorders (5th ed.); MINI-Kid—MINI International Neuropsychiatric Interview for Kids; CNS—Central Nervous System.

**Table 2:** Characteristics of the sample.

		ADHD *N* = 226 (%) *	non-ADHD *N* = 482 (%) *	ADHD vs. Non-ADHD *p*-Value (χ^2^ Test)
ADHD type	Combinet	171 (75.5)	-	-
	Attention-deficit disorder (ADD)	39 (17.3)	-	-
	Hyperactive/impulsive type (H/I)	16 (7.0)	-	-
Comorbid disorders	At least one additional diagnosed disorder	195 (86.3)	49 (10.2)	<0.000
	Learning disorders	102 (45.1)	22 (4.6)	<0.000
	Oppositional defiant disorder	70 (31.0)	-	-
	Speech disorders	56 (24.8)	27 (5.6)	<0.000
	Tic disorder	39 (17.3)	6 (1.2)	<0.000
	Conduct disorder	25 (11.1)	-	-
	Enuresis	51 (22.6)	-	-
	Anxiety disorders	10 (4.4)	-	-
	Mood disorders	7 (3.1)	-	-
Place of residence	Villages	50 (22.1)	35 (7.3)	<0.000
	City with less than 100,000 citizens	66 (29.2)	89 (18.4)	
	City with more than 100,000 citizens	110 (48.7)	358 (74.3)	
Mother’s level of education	Elementary	5 (2.4)	-	<0.000
	Vocational	32 (15.2)	54 (11.5)	
	Secondary	106 (50.5)	119 (25.3)	
	Higher	67 (31.9)	289 (63.3)	
Father’s level of education	Elementary	10 (4.8)	-	<0.000
	Vocational	69 (33.3)	103 (22.7)	
	Secondary	83 (40.1)	133 (29.3)	
	Higher	45 (21.7)	218 (48.0)	
Mother’s age at child birth	<25	67 (33.5)	89 (18.9)	<0.000
	25–35	111 (55.5)	346 (73.3)	
	>35	22 (11.0)	37 (7.8)	
Father’s age at child birth	<25	35 (18.0)	54 (11.7)	0.015
	25–35	126 (64.9)	350 (75.9)	
	>35	33 (17.0)	57 (12.4)	
Socioeconomic status	Low	15 (7.1)	16 (3.4)	<0.000
	Average	78 (36.8)	81 (17.5)	
	High	119 (56.1)	367 (79.1)	
Birth term	<37 weeks of gestation	29 (14.9)	32 (7.1)	<0.000
	between 37 and 42 weeks	138 (70.8)	417 (92.7)	
	>43 weeks of gestation	28 (14.3)	1 (0.2)	
The course of childbirth	the forces of nature	140 (66.3)	347 (72.0)	0.028
	Caesarean section	63 (29.9)	103 (21.4)	
	vacuum lift or forceps	8 (3.8)	32 (6.6)	
Birth body mass (g)	<2500	17 (8.5)	20 (4.2)	0.048
	2500–4000	164 (81.6)	390 (82.3)	
	>4000	20 (9.9)	64 (13.5)	
Apgar score	<4	9 (4.8)	14 (3.2)	0.001
	4–7	22 (11.7)	18 (4.1)	
	>7	157 (83.5)	411 (92.7)	
Weight status (IOTF)	Underweight	2 (1.08)	5 (1.05)	0.99645
	Normal weight	144 (77.42)	372 (77.99)	
	Overweight	29 (15.59)	71 (14.88)	
	Obesity	11 (5.91)	29 (6.08)	

* % calculated in relation to the answers given. International Obesity Task Force.

**Table 3:** Genotype and allele frequencies in ADHD and non-ADHD groups.

Gene Symbol	rs	Genotype	non-ADHD *N* (%)	ADHD *N* (%)	*p*-Value Genotypes	*p*-Value Alleles	OR (95% CI)	*p*-Value HWE
*ADRA2A*	rs553668	AA	5 (1.0)	3 (1.3)	0.655	0.365	1.169 (0.833–1.640)	0.471
		AG	99 (20.7)	53 (23.5)				
		GG	375 (78.3)	170 (75.2)				
***ADRA2A***	rs1800544	CC	317 (65.9)	128 (56.6)	0.056	**0.018**	0.725 (0.554–0.949)	0.973
		CG	146 (30.4)	86 (38.1)				
		GG	18 (3.7)	12 (5.3)				
*AGO1*	rs595961	AA	346 (72.5)	177 (78.3)	0.258	0.113	1.318 (0.935–1.858)	0.137
		AG	125 (26.2)	47 (20.8)				
		GG	6 (1.3)	2 (0.9)				
*ARL14*	rs1920644	CC	115 (23.9)	49 (21.9)	0.165	0.377	0.903 (0.721–1.131)	0.939
		CT	241 (50.1)	110 (49.1)				
		TT	125 (26.0)	65 (29.0)				
*BDNF*	rs6265	CC	341 (71.0)	157 (69.5)	0.728	0.841	0.968 (0.715–1.311)	0.834
		CT	125 (26.0)	64 (28.3)				
		TT	14 (3.0)	5 (2.2)				
*BHMT*	rs3733890	AA	40 (8.4)	25 (16.2)	0.337	0.147	1.194 (0.939–1.519)	0.786
		AG	201 (42.4)	100 (44.6)				
		GG	233 (49.2)	99 (44.2)				
*COMT*	rs4680	AA	136 (28.7)	49 (22.6)	0.242	0.152	0.847 (0.674–1.063)	0.579
		AG	226 (47.7)	112 (51.6)				
		GG	112 (23.6)	56 (25.8)				
*DBH*	rs2519152	CC	120 (25.2)	60 (26.5)	0.861	0.577	1.065 (0.851–1.332)	0.124
		CT	224 (46.9)	107 (47.4)				
		TT	133 (27.9)	59 (26.1)				
*DRD2*	rs1124491	AA	16 (3.3)	7 (3.1)	0.986	0.920	0.984 (0.733–1.320)	0.787
		AG	138 (28.8)	65 (28.8)				
		GG	326 (67.9)	154 (68.1)				
*DRD4*	rs1800955	CC	110 (23.3)	42 (18.8)	0.337	0.497	0.924 (0.737–1.160)	0.149
		CT	214 (45.2)	112 (50.2)				
		TT	149 (31.5)	69 (30.9)				
*FTO*	rs9939609	AA	101 (21.4)	40 (19.3)	0.158	0.527	1.077 (0.854–1.359)	0.490
		AT	216 (45.8)	111 (53.6)				
		TT	115 (32.8)	56 (27.1)				
*HTR1B*	rs6296	CC	267 (55.9)	123 (54.4)	0.612	1.000	1.000 (0.774–1.291)	0.445
		CG	173 (36.3)	89 (39.4)				
		GG	37 (7.8)	14 (6.2)				
*HTR2C*	rs518147	CC	114 (23.7)	72 (32.0)	<0.000	0.603	1.064 (0.840–1.347)	**<0.000**
		CG	92 (19.1)	12 (5.3)				
		GG	275 (57.2)	141 (62.7)				
*HTR2C*	rs3813929	CC	385 (80.5)	182 (80.5)	0.001	0.112	0.781 (0.576–1.060)	**<0.000**
		CT	52 (10.9)	10 (4.4)				
		TT	41 (8.6)	34 (15.1)				
*IPO11-HTR1A*	rs10042956	CC	429 (89.2)	197 (87.6)	0.523	0.777	0.967 (0.772–1.212)	0.111
		CT	52 (10.8)	28 (12.4)				
***KCNIP1***	rs1541665	CC	304 (63.7)	164 (73.2)	**0.044**	**0.015**	1.458 (1.074–1.979)	0.751
		CT	154 (32.3)	54 (24.1)				
		TT	19 (4.0)	6 (2.7)				
*MC4R*	rs17782313	CC	25 (5.3)	12 (5.6)	0.860	0.610	1.074 (0.812–1.421)	0.096
		CT	141 (30.5)	69 (32.4)				
		TT	297 (64.2)	132 (62.0)				
*MTHFR*	rs1801133	AA	45 (9.4)	16 (7.1)	0.548	0.314	0.880 (0.685–1.129)	0.629
		AG	194 (40.4)	90 (39.8)				
		GG	241 (50.2)	120 (53.1)				
***MTHFR***	rs1801131	GG	44 (9.2)	37 (16.4)	**0.013**	0.153	1.188 (0.937–1.505)	0.226
		GT	211 (43.9)	84 (37.2)				
		TT	225 (46.9)	105 (46.4)				
*MTR*	rs1805087	AA	287 (61.3)	136 (62.4)	0.830	1.000	1.008 (0.764–1.330)	0.910
		AG	161 (34.4)	71 (32.6)				
		GG	20 (4.3)	11 (5.0)				
*PIK3CG*	rs12667819	AA	100 (20.9)	39 (17.3)	0.515	0.442	0.915 (0.729–1.148)	0.110
		AG	215 (47.2)	108 (48.0)				
		GG	162 (33.9)	78 (34.7)				
*RSPH3*	rs183882582	AT	15 (3.2)	6 (2.7)	0.769	0.777	0.867 (0.334–2.252)	0.685
		TT	459 (96.8)	212 (97.3)				
***SLC1A3***	rs1049522	AA	220 (46.0)	80 (35.4)	**0.028**	**0.022**	0.763 (0.605–0.963)	0.735
		AC	201 (42.1)	115 (50.9)				
		CC	57 (11.9)	31 (13.7)				
***SLC6A2***	rs5569	AA	40 (8.4)	30 (13.3)	0.064	**0.029**	1.297 (1.026–1.640)	0.193
		AG	220 (45.9)	108 (47.8)				
		GG	219 (45.7)	88 (38.9)				
*SLC6A3*	rs463379	CC	26 (5.5)	8 (3.5)	0.322	0.841	1.026 (0.792–1.328)	0.054
		CG	184 (38.6)	98 (43.4)				
		GG	267 (55.9)	120 (53.1)				
*SLC6A4*	rs6354	GG	18 (3.8)	7 (3.1)	0.188	0.310	1.164 (0.868–1.560)	0.193
		GT	117 (34.7)	70 (31.3.2)				
		TT	338 (71.5)	147 (65.6)				
*SLC19A1*	rs1051266	CC	147 (30.6)	59 (26.2)	0.484	0.383	0.905 (0.723–1.132)	0.530
		CT	228 (47.4)	115 (51.1)				
		TT	106 (22.0)	51 (22.7)				
*SNAP25*	rs1051312	CC	28 (5.9)	11 (4.9)	0.847	0.887	0.980 (0.752–1.277)	0.997
		CT	170 (35.6)	83 (36.9)				
		TT	279 (58.5)	131 (58.2)				

OR—Odds Ratio (OR), 95% CI—95% Confidence Interval; HWE—Hardy–Weinberg equilibrium; rs—dbSNP Reference number. Significant effect was market with bold characters (*p*<0.05)

**Table 4:** List of genes with identified gene variants.

Gene Symbol	HGVSg	ALT	Consequence	Amino Acids	Existing VARIATION	Impact	PolyPhen	ADHD with/without QV for Gene	non-ADHD with/without QV for Gene	*P*-Value Fisher Exact Test
**(A) Detected Gene Variants in ADHD vs. Not Detect in Non-ADHD**										
*ADRA2A*	chr10:g.111078090G>C	C	MV	E/Q	rs753177273,COSV54528494	M	benign	2/47	0/46	0.263
*ADRA2A*	chr10:g.111078097C>A	A *	MV	P/Q	-	M	probably damaging			
*DBH*	chr9:g.133656524G>C	C *	MV, SRV	G/A	COSV67548769	M	probably damaging	1/48	0/46	0.516
*DYNC1H1*	chr14:g.102016033G>A	A	MV	A/T	rs766837403,COSV64136626	M	benign	1/48	0/46	0.516
***FBXL17***	chr5:g.108224144T>C	C	MV	K/E	rs141165823	M	benign	5/44	0/46	**0.033**
***FBXL17***	chr5:g.108380944C>A	A *	MV	A/S	-	M	benign			
***FBXL17***	chr5:g.108380956C>A	A *	MV	G/C	-	M	possibly damaging			
***FBXL17***	chr5:g.108381031_108381033dup	ACCG *	II	G/GG	rs906102490	M	-			
*MAP1A*	chr15:g.43527328A>C	C *	MV	E/A	-	M	probably damaging	1/48	0/46	0.516
*MTHFR*	chr1:g.11794399_11794400insAAA	CAAA *	II	-/F	-	M	-	1/48	0/46	0.516
*PCDH7*	chr4:g.30721475G>T	T	MV	C/F	rs757643187	M	benign	1/48	0/46	0.516
*RSPH3*	chr6:g.158999732A>C	C *	MV	I/R	rs1217221445	M	benign	1/48	0/46	0.516
*SCN2A*	chr2:g.165307860G>A	A *	MV	M/I	COSV51836794	M	probably damaging	1/48	0/46	0.516
*SEMA6D*	chr15:g.47771337G>A	A	MV	R/Q	rs766660850	M	probably damaging	2/47	0/46	0.263
*SEMA6D*	chr15:g.47771766T>C	C *	MV	L/P	rs540588380	M	possibly damaging			
*SPTBN1*	chr2:g.54599190C>G	G *	MV	R/G	rs915376910,COSV61693274	M	probably damaging	1/48	0/46	0.516
*TNRC6C*	chr17:g.78091580C>T	T *	MV	P/S	rs1302015314	M	benign	1/48	0/46	0.516
*ZNF536*	chr19:g.30548324_30548326del	A *	ID	RA/T	-	M	-	2/47	0/46	0.263
*ZNF536*	chr19:g.30549475A>C	C *	MV	T/P	rs1257346923	M	benign			
**(B) Detected Gene Variants in ADHD with EBW vs. Not Detect in Non-ADHD with NBW**										
*ADRA2A*	chr10:g.111078090G>C	C	MV	E/Q	rs753177273,COSV54528494	M	benign	1/13	0/44	0.241
*DYNC1H1*	chr14:g.102016033G>A	A	MV	A/T	rs766837403,COSV64136626	M	benign	1/13	0/44	0.241
*FTO*	chr16:g.53826140G>A	A	MV	A/T	rs79206939	M	benign	1/13	0/44	0.241
*MAP1A*	chr15:g.43527328A>C	C *	MV	E/A	-	M	probably damaging	1/13	0/44	0.241
*SEMA6D*	chr15:g.47771337G>A	A	MV	R/Q	rs766660850	M	probably damaging	1/13	0/44	0.241
*ZNF536*	chr19:g.30549475A>C	C *	MV	T/P	rs1257346923	M	benign	1/13	0/44	0.241

Rare variants with frequency: 0.009%, * very rare variant with frequency: 0.0009%, HGVSg—Human Genome Variation Society genomic nomenclature; ALT—Alternative variant; PolyPhen—Polymorphism Phenotyping (prediction of functional effects of human nsSNP and prediction of possible impact of an amino acid substitution on the structure and function of a human protein using straightforward physical and comparative considerations); QV—Qualifying variants; M—Moderate; MV—Missense Variant; II—Inframe Insertion; ID—Inframe Deletion; SRV—Splice Region Variant; EBW—Excessive Body Weight; NBW—Normal Body Weight; in bold—significant effect with *p* < 0.05.

## Data Availability

The data that support the findings of the study are available from the corresponding author, M.D.-W., upon reasonable request.

## Supplementary Materials

The following are available online at https://www.mdpi.com/article/10.3390/genes12091407/s1, Table S1: Information about the studied polymorphisms, Table S2: The list of genes with full names analyzed in SNP association and WES variant, Table S3: A unique collection of variants based on the Locus Reference Genomic (LRG), Table S4: List of rare variants with a frequency threshold of 0.009%, Table S5: List of very rare variants with a frequency threshold of 0.0009%, Table S6: Genotypes correlated with excessive body weight (EBW).

## Abbreviations

The following abbreviations are used in this manuscript

ADHD	Attention-Deficit/Hyperactivity Disorder
BMI	Body Mass Index
EBW	Excessive Body Weight
NBW	Normal Body Weight
IOTF	International Obesity Task Force
SNP	Single Nucleotide Polymorphism
WES	Whole Exome Sequencing
